# Characterization of transcriptional enhancers in the chicken genome using CRISPR-mediated activation

**DOI:** 10.3389/fgeed.2023.1269115

**Published:** 2023-10-25

**Authors:** Jeong Hoon Han, Hong Jo Lee, Tae Hyun Kim

**Affiliations:** ^1^ Department of Animal Science, The Pennsylvania State University, University Park, PA, United States; ^2^ Division of Animal Sciences, University of Missouri, Columbia, MO, United States; ^3^ The Huck Institutes of the Life Sciences, The Pennsylvania State University, University Park, PA, United States

**Keywords:** chicken, CRISPR activation, enhancer, FAANG, promoter, regulatory elements

## Abstract

DNA regulatory elements intricately control when, where, and how genes are activated. Therefore, understanding the function of these elements could unveil the complexity of the genetic regulation network. Genome-wide significant variants are predominantly found in non-coding regions of DNA, so comprehending the predicted functional regulatory elements is crucial for understanding the biological context of these genomic markers, which can be incorporated into breeding programs. The emergence of CRISPR technology has provided a powerful tool for studying non-coding regulatory elements in genomes. In this study, we leveraged epigenetic data from the Functional Annotation of Animal Genomes project to identify promoter and putative enhancer regions associated with three genes (*HBBA, IRF7*, and *PPARG*) in the chicken genome. To identify the enhancer regions, we designed guide RNAs targeting the promoter and candidate enhancer regions and utilized CRISPR activation (CRISPRa) with dCas9-p300 and dCas9-VPR as transcriptional activators in chicken DF-1 cells. By comparing the expression levels of target genes between the promoter activation and the co-activation of the promoter and putative enhancers, we were able to identify functional enhancers that exhibited augmented upregulation. In conclusion, our findings demonstrate the remarkable efficiency of CRISPRa in precisely manipulating the expression of endogenous genes by targeting regulatory elements in the chicken genome, highlighting its potential for functional validation of non-coding regions.

## 1 Introduction

Enhancers are DNA sequences bound by specific transcription factors and play an important role in regulating gene expression in a cell-specific manner (Heinz et al., 2015). They can be located upstream, downstream, or within the gene of interest and are responsive to external signals, enabling them to be activated or repressed (Heinz et al., 2015). Enhancers are particularly important in controlling gene expression in specific tissues, organs, and developmental stages, and it is increasingly relevant to understanding developmental and pathological processes (Claringbould and Zaugg, 2021). Previous research has primarily focused on the initiation of transcription by enhancers, leading to significant insights into the mechanisms governing tissue-specific and temporal gene expression (Arnone and Davidson, 1997). Furthermore, genetic variations in distant enhancers have been associated with various human Mendelian disorders, demonstrating the impact of enhancer sequences on disease susceptibility and phenotypic traits (Lettice et al., 2003). These findings highlight the importance of enhancers in gene regulation and their role in human health and disease. Therefore, identifying enhancers and their mechanisms of action are crucial areas of interest that may improve our current understanding of diseases and therapeutic approaches (Herz et al., 2014; Smith and Shilatifard, 2014).

The Encyclopedia of DNA Elements (ENCODE) Consortium has made significant contributions to our understanding of the human genome. In one of their studies, an estimated 399,124 regions with enhancer-like features were identified within the human genome (Consortium, 2012). This comprehensive research has provided valuable insights into the functional elements of the human genome, encompassing regulatory elements such as enhancers, promoters, and non-coding RNAs. By meticulously investigating this diverse repertoire of regulatory elements, researchers have gained important knowledge about the intricate regulatory landscape underlying gene expression. Furthermore, the successful collaboration between the ENCODE and epigenome consortia has demonstrated the effectiveness of jointly improving functional annotation (Consortium, 2012). The combination of this collaborative approach with the need to bridge the gap between genotype and phenotype serves as a strong motivation for the globally coordinated Functional Annotation of Animal Genomes (FAANG) project. FAANG, a widespread initiative, aims to provide comprehensive functional annotation of animal genomes, with a specific focus on regulatory elements (Kern et al., 2021). Enhancers are of particular interest within the FAANG project, as they play a crucial role in regulating gene expression across different species. Researchers are developing methods to identify and characterize enhancers in different species, including humans and model organisms such as mice and chickens (Uchikawa et al., 2004; Sethi et al., 2020). Nonetheless, despite significant progress, the understanding of the relationship between enhancer sequences from different evolutionary origins and their regulatory functions, as well as the interplay between gene regulatory functions and sequences from distinct evolutionary periods within complex enhancers, remains limited (Fong and Capra, 2022).

The Clustered Regularly Interspaced Short Palindromic Repeats (CRISPR)/CRISPR-associated nuclease 9 (Cas9) system is a revolutionary gene editing tool that has transformed our ability to study and manipulate genetic material (Jinek et al., 2012; Cong et al., 2013; Mali et al., 2013). Originally discovered in microbial immune systems as a DNA degradation mechanism, CRISPR/Cas9 is now recognized as a powerful gene editing tool (Mali et al., 2013; Doudna and Charpentier, 2014). Additionally, it enables transcriptional activation and repression through the use of nuclease-dead Cas9 (dCas9) and guide RNA (gRNA) (Jinek et al., 2012; Hilton et al., 2015; Kiani et al., 2015; Dominguez et al., 2016). CRISPR activation (CRISPRa) utilizes dCas9 and gRNA to regulate gene expression and is highly efficient and specific in both *in vitro* and *in vivo* studies (Kampmann, 2018). By delivering modified dCas9 effectors and gRNA via plasmids or viral vectors, genes can be activated or repressed (Forstneric et al., 2019; Black et al., 2020; Di Maria et al., 2020). Transcriptional activator domains such as VP64 (Maeder et al., 2013), and VP64-p65-Rta (VPR) (Chavez et al., 2015), have been employed to enhance gene activations. Unlike traditional CRISPR/Cas9 approaches, CRISPRa does not break DNA but recruits transcriptional machinery to specific sites, thereby improving gene expression (Konermann et al., 2015). This approach has proven valuable in identifying active enhancers and can be scaled for genome-wide screening across different species (Chavez et al., 2016; Casas-Mollano et al., 2020). By introducing specific gRNAs targeting promoters or enhancers, CRISPRa enables concentration-dependent and reversible gene regulation, on gene regulatory mechanisms (Dai et al., 2021). CRISPRa has emerged as a powerful tool for investigating the role of potential enhancers in gene regulation (Panigrahi and O'Malley, 2021). For example, it has been successfully utilized in human cells to identify enhancers involved in T cell activation (Schmidt et al., 2022). Similarly, a doxycycline-inducible dCas9-VPR was expressed in mouse embryonic stem cells, resulting in an inducible CRISPRa system that enables concentration-dependent and reversible activation of target genes via specific gRNAs targeting promoters or enhancers (Dai et al., 2021). Although the use of CRISPRa has been utilized in many species, the use of this technology in chicken tissues and cell lines remains understudied.

The dCas9-VPR and dCas9-p300 CRISPR activation systems were utilized, known for their effectiveness in inducing gene expression in other species (Casas-Mollano et al., 2020; Dominguez et al., 2022). The dCas9 protein, which lacks endonuclease activity, can target specific genomic regions by binding to a DNA sequence via its gRNA (Qi et al., 2013). The dCas9-VPR system recruits proteins involved in transcription to the target DNA region and results in increased and specific gene expression by using a fusion protein between the dCas9 protein and two transcriptional activation domains, VP64, p65, and Rta (VPR) (Chavez et al., 2015). The dCas9-p300 system modifies the nearby chromatin structure and increases gene expression by utilizing a fusion protein between the dCas9 protein and the transcriptional co-activator p300 histone acetyltransferase (Hilton et al., 2015). The p300 histone acetyltransferase is a transcriptional co-activator that plays a critical role in regulating gene expression (Ghosh and Varga, 2007). It works by adding acetyl groups to histone proteins, which helps to loosen the tightly packed chromatin structure and facilitate access to the DNA by the transcriptional machinery (Sterner and Berger, 2000; Miller and Grant, 2013). This process is known as histone acetylation, and it is an important mechanism for controlling gene expression in response to various signals and stimuli (Ghosh and Varga, 2007). By fusing the dCas9 protein to p300, the dCas9-p300 system can selectively target and activate specific genes in the genome, offering a powerful tool for studying gene regulation and potentially treating genetic diseases (Klann et al., 2017). This study selected the dCas9-VPR and dCas9-p300 systems based on their demonstrated effectiveness in inducing gene expression in other species, with the aim of addressing the challenges associated with developing efficient CRISPR/Cas9 systems for avian species.

Chickens are not only an important agricultural species but also serve as excellent model organisms for studying developmental biology and immune responses (Burt, 2005). Understanding the mechanisms that regulate gene expression in chickens can provide valuable insights into various biological processes and disease mechanisms. Gene regulation lies at the core of this understanding, as it orchestrates the activation or silencing of genes, thereby exerting precise control over the production of proteins and other vital molecules within cells (Miyamoto and Gurdon, 2013). By studying gene regulation in chickens, researchers can gain insights into important biological processes such as development, growth, and immune response. In addition, the application of CRISPRa in chickens remains relatively unexplored, presenting a promising avenue for investigating and identifying potential enhancers in avian species. The DF-1 cell line, derived from spontaneously immortalized chicken fibroblasts, has emerged as an invaluable model system for avian viral research and recombinant protein expression (Himly et al., 1998).

In our previous study (Chapman et al., 2023), we demonstrated the potent capability of the CRISPRa system in chickens to achieve targeted upregulation of endogenous genes by activating promoter regions. In this research, our objective was to functionally validate enhancers within the chicken genome employing the CRISPRa system. As a proof of principle, we employed epigenomic data from the FAANG dataset to identify putative enhancer regions of three genes in the chicken genome and utilized the CRISPRa toolkit, specifically dCas9-VPR and dCas9-p300, for validation of transcriptional enhancers.

## 2 Materials and methods

### 2.1 Cell culture and cell line establishment

The chicken fibroblast cell lines (DF-1) (CRL-12203; American Type Culture Collection (ATCC), Manassas, VA, United States) were utilized to generate the CRISPRa cell line (SP-dCas9-VPR and pcDNA-dCas9-p300 Core) as described in a previous study (Chapman et al., 2023). These cell lines were cultured and sub-passaged in Dulbecco’s minimum essential medium (DMEM; Hyclone, Logan, UT, United States) supplemented with 1x antibiotic-antimycotic (ABAM; Thermo Fisher Scientific, Waltham, MA, United States) and 10% fetal bovine serum (FBS; Thermo Fisher Scientific). Additionally, the cells were subjected to continuous drug selection using Geneticin Selective Antibiotic (G418, 300 μg/mL) (Thermo Fisher Scientific). The CRISPRa cell lines were maintained at 37°C with 5% CO_2_ and 60%–70% relative humidity.

### 2.2 Identification of promoter and potential enhancer regions in chicken genome

The study utilized chicken genomic annotation and regulatory element prediction data from the Functional Annotation of Animal Genomes (FAANG) dataset to identify potential promoter and enhancer regions in the chicken genome (Kern et al., 2021). This included three histone modifications (H3K4me3, H3K27ac, and H3K4me1) obtained through chromatin immunoprecipitation sequencing (ChIP-seq), as well as DNase I hypersensitive site sequencing (DNase-seq) data and RNA sequencing (RNA-seq) data. The DF-1 RNA-seq dataset, consisting of SRR18704488, SRR18704497, and SRR18704496, was obtained from the NCBI Sequence Read Archive (SRA) under the project accession number PRJNA825282. Integrative Genome Viewer (IGV, version 2.12.3) was used for data visualization (Thorvaldsdottir et al., 2013; Robinson et al., 2023).

To investigate tissue-specific gene expression, we compared RNA-seq data between the eight tissues used in Kern et al., for each candidate gene. To identify potential promoter regions in the chicken genome, DNase hypersensitivity (DHS) and H3K4me3 were examined near the known transcription start site of each gene. To identify potential enhancer regions in the chicken genome, ChIP-seq data for two histone markers, H3K4me1 and H3K27ac, and DNase-seq data were visually examined across the predicted regulatory elements near each gene. Putative enhancer regions were called when the peaks for these markers overlapped at the predicted regulatory elements annotated by Kern et al. (2021).

### 2.3 gRNA vector cloning and design for promoter and enhancer region

gRNAs were expressed by gRNA expression vector used in our previous study (Chapman et al., 2023). It included a gRNA scaffold driven by the human U6 promoter and a puromycin resistant gene. BbsI restriction enzyme digestion and ligation were used to clone gRNAs targeting each gene or mock controls into the vector (Ran et al., 2013).

The gRNAs were designed to target the promoter and putative enhancer regions of three genes: *HBBA, IRF7*, and *PPARG*. Four gRNAs were designed for the targeting promoter region of *HBBA* and four gRNAs for the two enhancer regions. In our previous study (Chapman et al., 2023), gRNAs were designed specifically for the promoter regions of *IRF7* and *PPARG* genes. Additionally, three gRNAs were designed for each distinct enhancer region associated with *IRF7*, while four distinct enhancer regions were targeted with three gRNAs each for *PPARG*. The design of gRNAs was done by CHOPCHOP algorithm (https://chopchop.cbu.uib.no/) (Montague et al., 2014). In addition, a mock control was included in the study, for which three gRNAs that did not match the chicken genome were developed. The gRNA and oligonucleotide sequences are listed in Supplementary Table S1.

### 2.4 gRNA transfection

To activate the promoter region, individual gRNA vectors were prepared and then transfected into established CRISPRa cell lines (dCas9-VPR and dCas9-p300) using Lipofectamine 2000 (Thermo Fisher Scientific). Cells were transfected when they reached 70% confluence in a 12-well plate. For transfection, Lipofectamine 2000 reagent (3 µL) was diluted in 100 µL of Opti-MEM (Thermo Fisher Scientific) reagent. Simultaneously, 3 µg of total plasmid DNA was diluted in 100 µL of Opti-MEM. The diluted DNA was then combined with the diluted Lipofectamine 2000 reagent in a 1:1 ratio. The mixture was incubated for 5 min to allow for complex formation. Subsequently, the DNA-Lipofectamine complex was added to the CRISPRa cell lines for transfection. To activate the enhancer regions, both promoter and enhancer-targeting gRNAs co-transfection was executed resulting in a combined total of 3 µg of gRNAs. For the *HBBA* gene, a co-transfection approach involved combining 1 µg of promoter-gRNA and 500 ng each of four enhancer-gRNAs targeting each candidate enhancer. For *IRF7* activation, 1.5 µg of promoter-gRNA was paired with 500 ng each of three gRNAs for each enhancer targeting gRNA. For *PPARG* activation, 1 µg of promoter-gRNA vector and 2 µg (500 ng for each) enhancer-gRNAs were used.

After a 24-h transfection, cells were subjected to 48 h puromycin treatment (1 μg/mL) (Thermo Fisher Scientific) and harvested 72 h post-transfection. RNA extraction was performed using the Direct-Zol RNA Miniprep kit (Zymo Research, Irvine, CA, United States) following the manufacturer’s protocol.

### 2.5 Quantitative real-time polymerase chain reaction (qRT-PCR)

To synthesize cDNA, 1 µg of total RNA was used with the High-Capacity cDNA Reverse Transcription Kit (Thermo Fisher-Invitrogen). The reverse transcription master mix was generated using the manufacturer’s protocol, including RNase Inhibitor. For qRT-PCR, the PowerUp SYBR Green Master Mix (Thermo Fisher-Invitrogen) was used according to manufacturer’s protocol. Cycling conditions included 50°C for 2 min, 95°C for 2 min, 40 cycles of 95°C for 15 s, and 60°C for 1 min, followed by a melting cycle. The expression level of the housekeeping gene *GAPDH* was used for gene expression normalization, and the *ΔΔ*Ct method was applied (Livak and Schmittgen, 2001). The primer sequences utilized in this study can be found in Supplementary Table S2. In this study, at least three biological repetitions were conducted for all qRT-PCR experiments. Two-tailed t-tests were performed and visualized using GraphPad Prism (GraphPad Software, CA, United States).

## 3 Results

### 3.1 Identification of chicken β-globin locus control region

**FIGURE 1:**
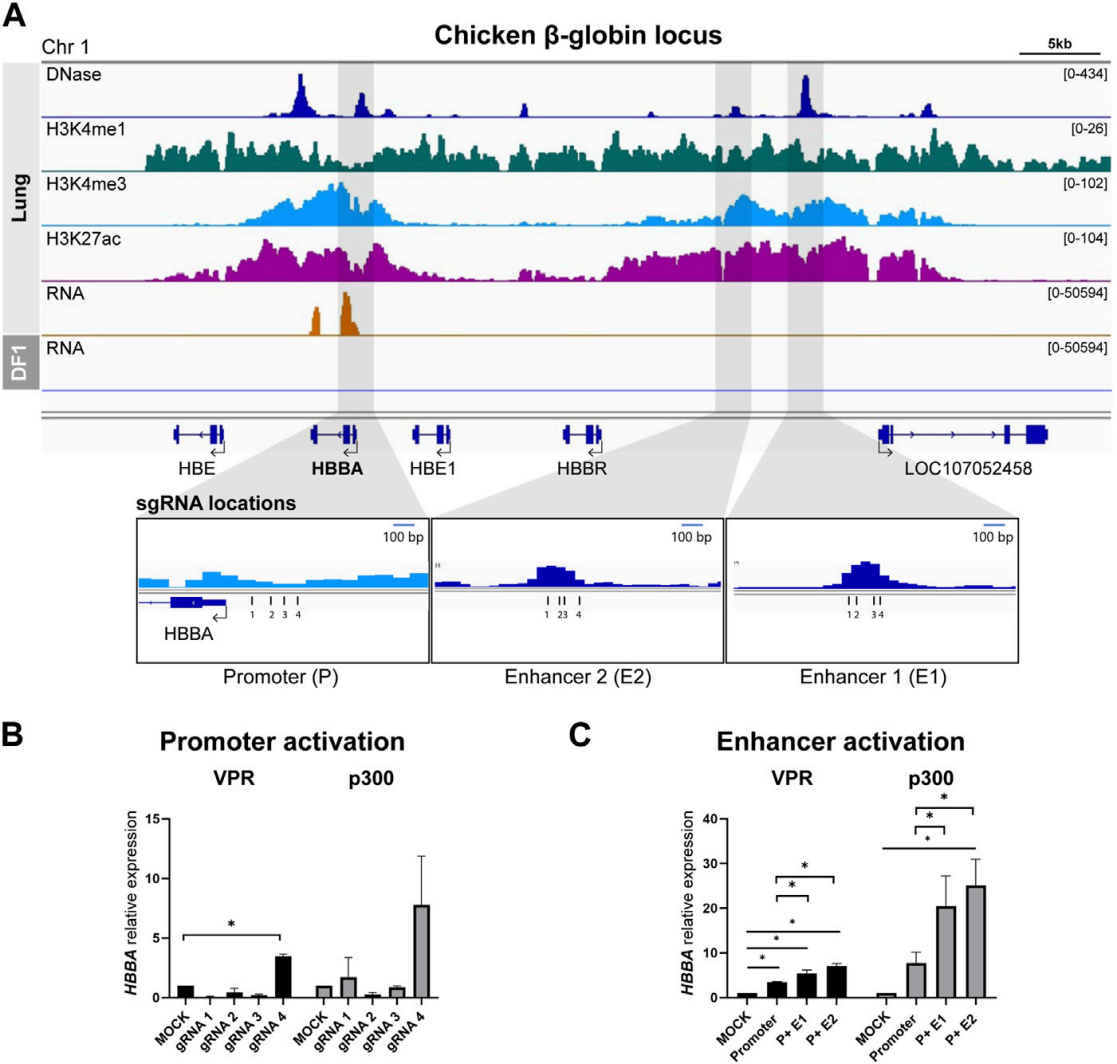
Activation of predicted cis-Regulatory elements in chicken β-globin locus **(A)** Representative genome browser track of the chicken β-globin locus in lung tissue from Kern et al., 2021 and DF-1. The grey marked regions correspond to the predicted cis-regulatory elements (cREs) and gRNA locations are indicated. **(B)** Activation of *HBBA* upon activation of promoter region using four different gRNAs. **(C)** The expression of *HBBA* upon dCas9-VPR and dCas9-p300 activation of the predicted cREs in DF-1 cells. The data are presented as mean ± SEM. Statistical significance is indicated by *(*p* < 0.05, Student’s t-test).

We used lung tissue data to identify the chicken ortholog of the well-studied human β-globin locus control region (HBB-LCR), locating it on chromosome 1 with a similar epigenomic landscape (Figure 1A). Among the four genes within the chicken β-globin locus (*HBE*, *HBBA*, *HBE1*, and *HBBR*), *HBBA* exhibited significantly higher expression in the RNA-seq data. Consequently, we selected the *HBBA* gene for subsequent analyses. We identified the promoter region in close proximity to the transcription start site (TSS) and successfully delineated two upstream distal enhancer regions: E1 (−14.5 kb from the *HBBA* TSS) and E2 (−12.1 kb from the *HBBA* TSS). We designed four gRNAs to target the promoter and each distal putative enhancer region of the *HBBA* gene (see Figure 1A).

First, we tested and selected the most effective gRNA to activate the promoter from among the four designed gRNAs. In the dCas9-VPR cell line, we observed a significant increase in the relative expression of *HBBA* mRNA when using promoter-targeting gRNA4 compared to the mock control (Figure 1B). A similar trend was observed in the dCas9-P300 cell line, although it did not reach statistical significance (Figure 1B). Based on this result, we used promoter-gRNA4 in subsequent enhancer activation experiments.

To investigate the function of the putative enhancers, we co-activated both the promoter and enhancers, simulating the mechanism of enhancer action. In the dCas9-VPR cell line, co-activation of the promoter and E1 resulted in a significant increase in the relative abundance of *HBBA* mRNA, with a 5.3-fold increase compared to the mock control and a 1.55-fold increase compared to promoter activation alone (Figure 1C). Co-activation of the promoter with E2 resulted in a higher increase in expression, with a significant 7.1-fold upregulation compared to the mock control and a 2.0-fold increase compared to the sole activation of the promoter (Figure 1C). A similar trend was observed in the dCas9-p300 based activation, where we observed a significant augmentation of expression due to the co-activation of the promoter and E2, resulting in a 25.1-fold increase in the relative expression of *HBBA* compared to the mock control and a 3.2-fold increase compared to the promoter activation (Figure 1C). Activation of the promoter and E1 in dCas9-p300 cells also resulted in a substantial increase in *HBBA* expression, but the magnitude of this upregulation was less pronounced than that observed with the promoter and E2 co-activation (Figure 1C).

### 3.2 Identification of chicken *IRF7* enhancers

**FIGURE 2:**
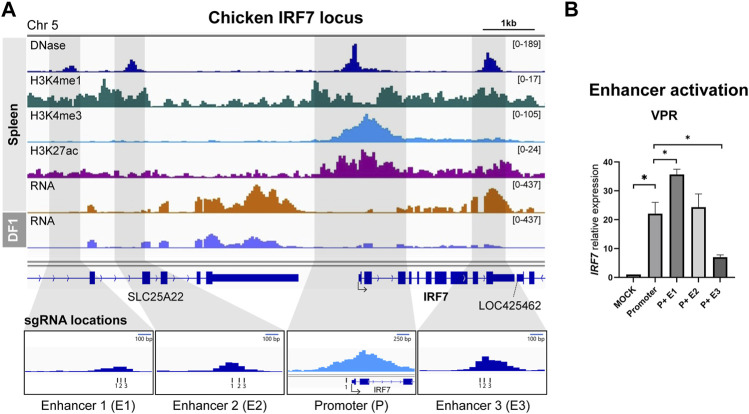
Activation of predicted cis-regulatory elements in the chicken *IRF7* locus **(A)** Representative genome browser track of chicken *IRF7* locus in spleen from Kern et al., 2021 and DF-1. The grey marked regions correspond to the predicted cis-regulatory elements (cREs) and gRNA locations are indicated. **(B)** The expression of *IRF7* upon dCas9-VPR activation of the predicted cREs in DF-1 cells. The data are presented as mean ± SEM. Statistical significance is indicated by *(*p* < 0.05).

We employed an identical methodology to the one used for identifying the chicken HBB-LCR to uncover cREs of *IRF7*, as spleen tissue exhibited notably elevated expression of *IRF7* in RNA-seq analyses (Figure 2A). In relation to the TSS, E1 and E2 were positioned at distances of 6.2 and 4.8 kb upstream, respectively, while E3 overlapped with the final exon, situated 2.8 kb downstream of the TSS (Figure 2A). We utilized the gRNA sequence identified for *IRF7* promoter activation from our previous study (Chapman et al., 2023), and designed separate gRNAs for targeting enhancers (Figure 2A). The activation of *IRF7* cREs was exclusively conducted using the dCas9-VPR system, as the use of dCas9-p300 for *IRF7* promoter activation did not lead to increased gene expression in our earlier research (Chapman et al., 2023) (Figure 2B).

The activation of the promoter led to a significant increase in *IRF7* expression, as anticipated, showing a 22.0-fold rise compared to the mock control (Figure 2B). Co-activation of putative enhancers yielded varying results. P + E1 activation resulted in a substantial 35.7-fold upregulation compared to the promoter alone, whereas P + E2 did not exhibit a significant difference compared to promoter activation, and P + E3 showed a significantly lower expression level than promoter activation (Figure 2B). Hence, it is possible that the E1 region functions as an enhancer for the chicken *IRF7* gene in the fibroblast cell line, while E2 and E3 may not.

### 3.3 Identification of chicken *PPARG* enhancers

**FIGURE 3:**
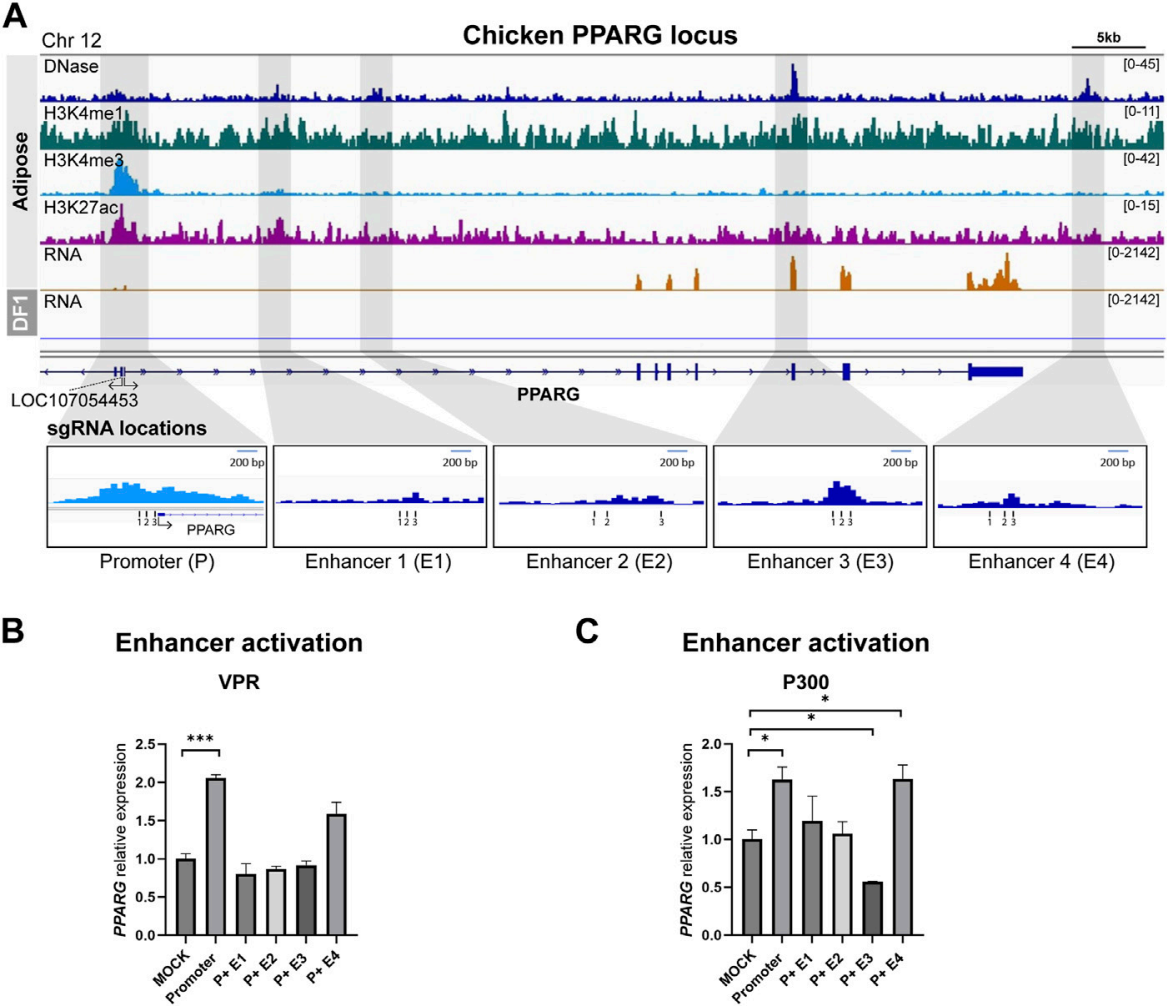
Activation of predicted cis-regulatory elements in the chicken *PPARG* locus **(A)** Representative genome browser track of chicken *PPARG* locus in adipose tissue from Kern et al., 2021 and DF-1. The grey marked regions correspond to the predicted cis-regulatory elements (cREs) and each gRNA locations are indicated. **(B)** The expression of *PPARG* upon dCas9-VPR activation of the predicted cREs in DF-1 cells. **(C)** The expression of *PPARG* upon dCas9-p300 activation of the predicted cREs in DF-1 cells. The data are presented as mean ± SEM. Statistical significance is indicated by *(*p* < 0.05) or *** (*p* < 0.001).

To identify potential enhancers of *PPARG*, we utilized data from adipose tissue and identified four putative enhancers located downstream of the transcription start site (TSS). Among these four putative enhancers, two were situated in the first intron, the third overlapped with the fifth exon, and the last one was positioned 5.7 kb downstream of the 3′UTR (Figure 3A). The activation results from both the dCas9-VPR and dCas9-p300 cell lines showed that while targeting the *PPARG* promoter alone resulted in a significant increase in gene expression, co-activation of the promoter and enhancers did not lead to higher levels of upregulation compared to the promoter gRNA transfection (Figures 3B, C). In some cases, the addition of enhancer-targeting gRNAs resulted in a significant decrease in *PPARG* expression (Figures 3B, C).

## 4 Discussion

The primary objective of this study was to identify enhancer regions within the chicken genome using CRISPR-based transcriptional activation systems. We integrated publicly available functional genomics data to identify putative enhancer regions *in silico* and molecularly validated regulatory element annotations. While enhancer regions have been extensively studied in various species, it is important to note the limited research dedicated to discovering and functionally validating enhancers in the chicken genome (Halstead et al., 2020; Kern et al., 2021). Methods for exploring enhancer regions involve analyzing ChIP-seq data, focusing on specific histone markers, including H3K27ac, H3K4me1 and H3K4me3 (Spicuglia and Vanhille, 2012). RNA-Seq stands out as a highly accurate approach for quantifying transcripts (Wang et al., 2009), and DNase-seq can identify regions of open chromatin that mark diverse classes of cREs (Li et al., 2019).

The catalogs of regulatory elements in humans and mice have played a crucial role in identifying genetic variants linked to health and disease (Consortium, 2004; Consortium, 2012; Maurano et al., 2012; Mouse et al., 2012; Roadmap Epigenomics et al., 2015). Recently, the ENCODE phase three project has highlighted the significant importance of functional elements, emphasizing their relevance in evolutionary biology, human medicine, and the precise prediction of genotype-to-phenotype correlations with greater detail and accuracy In the realm of animal genomics, namely, FAANG community, efforts have been made to identify regulatory elements in economically important species. Initial work in this area has identified 29,526 predicted interactions between regulatory elements and genes in the chicken genome using functional genomics data from multiple tissue types (Kern et al., 2021). However, a significant portion of these regulatory elements does not appear to exert their regulatory influence on nearby genes, making it challenging to determine their precise functions and establish definitive connections with their target genes. Validating such annotations requires the careful use of molecular tools to confirm their regulatory roles.

Additionally, previous studies have shown the effectiveness of dCas9-p300 in inducing epigenetic modifications at the target gene locus, resulting in long-lasting effects on gene expression (Dominguez et al., 2022). Within a mouse model, it was observed that the dCas9-VPR system exhibited the most remarkable capacity for elevating the expression levels of target genes, surpassing the efficacy of alternative CRISPR/dCas9 systems under investigation (Dominguez et al., 2022). Furthermore, this system was used with a trans enhancer sequence designed to activate Myod1 expression and induce muscle regeneration in mice (Xu et al., 2019). Also, dCas9-p300 induced transcription from gRNA-targeted promoters and induced the expression of distal globin genes when targeting the HS2 enhancer in the β-globin locus control region (Zlotorynski, 2015). The results also showed that the acetyltransferase activity of dCas9-p300 Core was necessary for transcription activation, and that the deposition of H3K27ac marks was significantly enriched at the targeted enhancer and promoters (Zlotorynski, 2015). Hence, we utilized dCas9-p300 and dCas9-VPR to pinpoint and study enhancer regions within the chicken genome.

The HBB-LCR cluster, located approximately 50–70 kb upstream of the HBB genes in both human and murine genomes, is a well-known enhancer region crucial for enhancing the expression of globin genes (Hardison, 2012). Due to the limited information available about enhancers in the chicken genome, our initial efforts focused on identifying the chicken counterpart of the mammalian HBB-LCR, and we successfully identified potential enhancer regions that has similar epigenomic landscape with four hypersensitive sites within the β-globin locus. We chose two upstream regions (E1 and E2) to test based on the DHS peak intensity and our study suggests that both regions are functional enhancers.

To further demonstrate the feasibility of enhancer activation, we also utilized two transcription factor genes, *IRF7* and *PPARG*, both of which we successfully upregulated in response to targeted promoter activations using CRISPRa systems in our previous study (Chapman et al., 2023). While we successfully pinpointed the functional enhancer of chicken IRF7, it is worth noting that certain predicted regulatory regions in the epigenomic data did not exhibit enhancer activity in this investigation. Interestingly, some of these regions exhibited decreased expression upon targeted activation. These intriguing findings suggest the possibility of undiscovered enhancer regions or the potential for these putative enhancers to act on entirely different genes. Additionally, it is noteworthy that we observed a decrease in gene expression, especially when gRNAs targeted exon regions, while using CRISPRa effectors to target potential enhancers located within the gene itself, as demonstrated in both the PPARG and IRF7 experiments. This decrease in expression may result from potential interference with the transcriptional process. Therefore, these observations highlight the need for alternative strategies when validating enhancer regions found within gene bodies (Karlson et al., 2021). Similar findings have been reported in previous studies, where CRISPR-directed gene editing targeting exons resulted in exon skipping and alterations in gene expression (Banas et al., 2022). It is also known that different cell lines can have different transcriptional regulation mechanisms, and the regulatory regions of a gene may differ depending on the cell line being used (Kang et al., 2020; Chua et al., 2022).

Our study revealed significant variations in activation efficiency between two different activators. Specifically, the dCas9-P300 system outperformed the dCas9-VPR system for *HBBA* activation, whereas substantial expression changes in *IRF7* were primarily achieved using the dCas9-VPR system. These variations in effectiveness likely stem from differences in their mechanisms of action, with p300 functioning as a histone acetyltransferase and VPR primarily acting as a transcription factor. One potential contributing factor to these divergent outcomes could be the distinct chromatin environment at the targeted loci within the native chromatin context (Konermann et al., 2015). For instance, the chromatin landscape at the enhancer regions may have favored dCas9-P300 activation for *HBBA*, resulting in a significant increase in expression compared to the control group. Conversely, the chromatin environment in DF-1 cells may have been more suitable for dCas9-VPR, leading to the observed expression changes in *IRF7*. Utilizing additional functional genomics data for cell selection could enhance the informed decision-making process, aligning with previous research suggesting that the dCas9-VPR system may be more effective in specific cell types (Wu et al., 2023).

While the CRISPRa system, as demonstrated in our study, holds promise in identifying promoters and enhancers through targeted activation, it is essential to acknowledge inherent limitations. Firstly, the epigenetic state of enhancers is a significant factor, as enhancers within closed chromatin regions may face accessibility constraints, potentially limiting their ability to activate transcription (He et al., 2014). Secondly, not all enhancers are equally effective, and their functionality depends on various factors, including sequence variations, the presence of specific transcription factor binding sites, and the chromatin context Thirdly, the design of experiments, including choices related to promoter selection and gRNA localization, significantly influences activation efficacy. Additionally, selecting specific cell types for specific genes may introduce limitations impacting overall outcomes (Panigrahi and O'Malley, 2021; Trapnell, 2015).

In summary, we empirically validated transcriptional enhancers within the chicken genome based on regulatory element annotations from the FAANG data. This study highlights the promising potential of the CRISPRa-based gene activation approach for identifying and elucidating enhancer regions in various chicken genes. These findings could significantly contribute to our understanding of the complex regulatory mechanisms governing gene expression within the chicken genome. Furthermore, our investigation underscores the effectiveness of the CRISPR-based activation system in identifying enhancer elements within the avian genome that could be used for comparative genomics studies. It is essential to assess the applicability of the CRISPR toolkit across diverse chicken cell lines originating from distinct lineages to enable a more comprehensive exploration of context-specific biology. Additionally, the development of a high-throughput platform tailored to the avian system can help reveal the functional roles of genome-wide regulatory elements in the avian context, further enhancing our understanding of avian genomics.

## Data Availability

The datasets presented in this study can be found in online repositories. The names of the repository/repositories and accession number(s) can be found in the article/Supplementary Material.
